# Treating dental crowding with mandibular incisor extraction in an Angle Class I patient

**DOI:** 10.1590/2176-9451.20.3.101-108.bbo

**Published:** 2015

**Authors:** Gislana Braga Machado

**Affiliations:** 1 Specialist in Orthodontics, Universidade de São Paulo (USP), Bauru, São Paulo, Brazil. Specialist in Temporomandibular Disorders and Orofacial Pain, Conselho Federal de Odontologia (CFO). Diplomate of the Brazilian Board of Orthodontics (BBO)

**Keywords:** Angle Class I malocclusion, Tooth extraction, Incisor

## Abstract

Mandibular dental crowding often encourages patients to seek orthodontic treatment. The orthodontist should decide between protrusion of incisors or decrease in dental volume so as to achieve proper alignment and leveling. The present study reports the treatment of an Angle Class I malocclusion adolescent female brachyfacial patient with severe mandibular dental crowding, increased curve of Spee and deep overbite. The patient was treated with extraction of a mandibular incisor. This case was presented to the Brazilian Board of Orthodontics and Dentofacial Orthopedics (BBO) as a requirement for the title of certified by the BBO.

## Abstract (pt)

O apinhamento dentário inferior, com frequência, motiva os pacientes a buscarem tratamento ortodôntico. Ao ortodontista cabe a decisão entre protruir os incisivos ou diminuir o volume dentário, a fim de obter o adequado alinhamento e nivelamento. O presente relato aborda o tratamento de uma má oclusão Classe I de Angle, em paciente adolescente, sexo feminino, braquifacial, com severo apinhamento na arcada inferior, curva de Spee e sobremordida aumentadas, e tratada com exodontia de um incisivo inferior. Esse caso clínico foi apresentado à Diretoria do Board Brasileiro de Ortodontia e Ortopedia Facial (BBO) como parte dos requisitos para a obtenção do título de Diplomado pelo BBO.

## INTRODUCTION

The present study reports the case of a Caucasian female patient who sought orthodontic treatment at the age of 12 years and 5 months, with good general health and chief complaint of mandibular dental crowding. Her medical records were not significant, while her dental records revealed trauma with a minor fracture on the incisal edge of her right maxillary central incisor. 

## DIAGNOSIS

**Figure 1.**
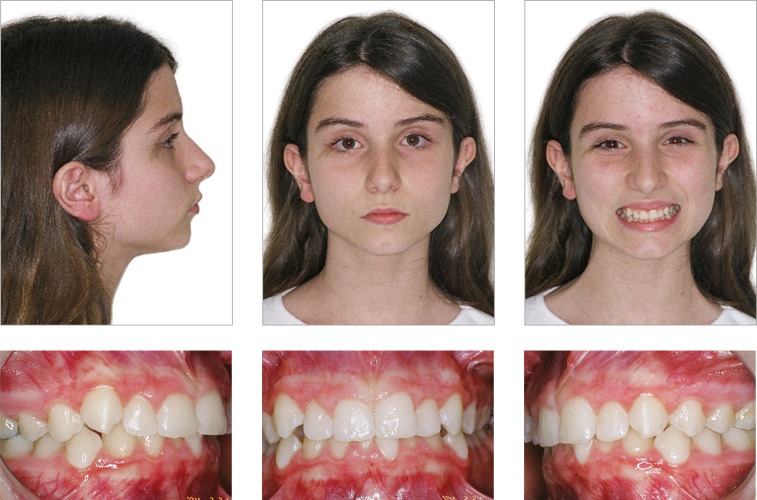
Initial facial intraoral photographs.

**Figure 2.**
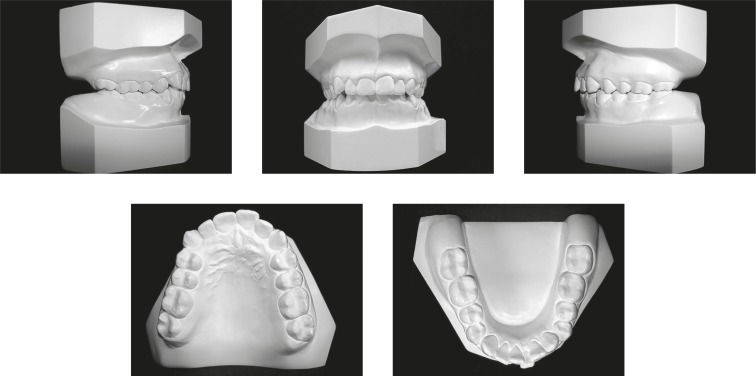
Initial casts.

**Figure 3.**
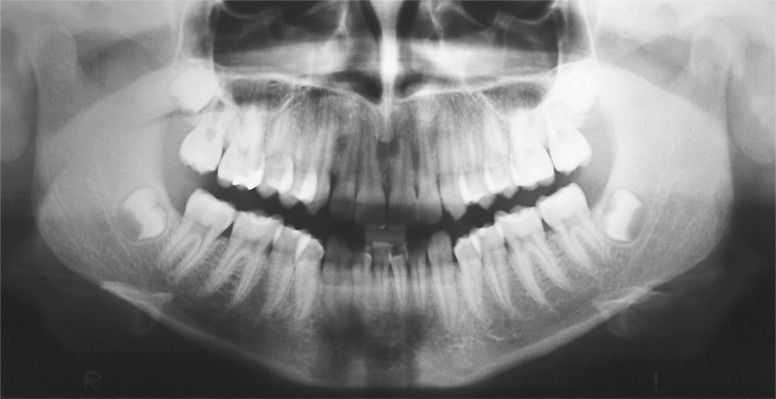
Initial panoramic radiograph.

**Figure 4.**
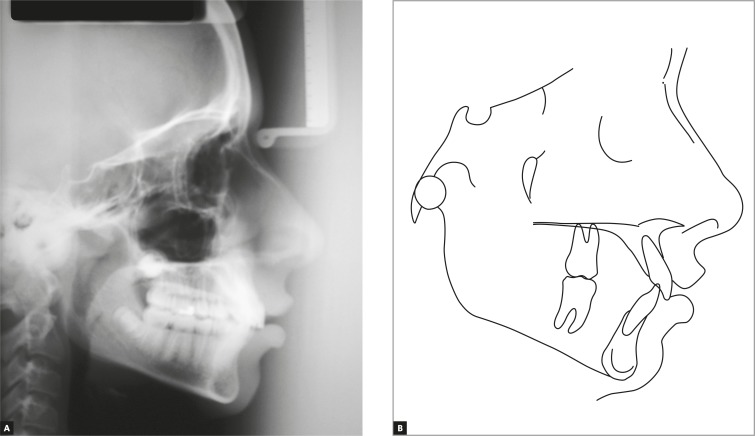
Initial lateral cephalogram (A) and cephalometric tracing (B). A B

Frontal-view facial analysis revealed balanced facial thirds and slightly everted lower lip. Lateral-view analysis revealed a convex profile, mild mandibular deficiency and mild bimaxillary lip protrusion (Fig 1). From a dental perspective, the patient was classified as Angle Class I, with 3.5-mm overbite, 1-mm overjet, increased curve of Spee, 3.5-mm upper crowding and 7-mm lower crowding (Figs 1, 2). Panoramic radiograph revealed the presence of all teeth with root integrity (Fig 3). Cephalometrically (Fig 4 and Table 1), the patient had a Class II skeletal brachyfacial pattern, with mandibular deficiency (SNA = 78^o^; SNB = 73.5^o^; ANB = 4.5^o)^, well-positioned maxillary incisors and tipped as well as protruded mandibular incisors ( 1.NB = 30^o^ and 1-NB = 7 mm). 

## TREATMENT PLAN

Although mandibular dental crowding, protrusion and tipping of mandibular incisors and the need to correct the curve of Spee suggested that extraction of premolars was necessary, patient's horizontal facial growth pattern and mild maxillary dental crowding contraindicated them, as they could result in profile flattening.^1 ^Nevertheless, despite potential mandibular interproximal stripping,^2^ treatment without teeth extractions could lead to excess tipping of mandibular incisors, thus hindering lip seal and stability. For this reason, the possibility of locally solving the problem with extraction of a mandibular incisor was considered, despite being aware that overjet could not be incresed.^1 ^To this end, it was important to avoid excess tipping of maxillary incisors, increase the crown of the remaining mandibular central incisor and wait for anterior mandibular protrusion to happen as a result of growth.

Based on this proposal, treatment plan was developed with a view to keeping the Class I molar and canine relationship, adapting lateral and protrusive guidances, aligning maxillary teeth so as to avoid increase in protrusion, aligning and leveling mandibular teeth so as to correct the curve of Spee and reduce overbite, and keeping protrusion and tipping of mandibular incisors unchanged so as to preserve passive lip seal. To this end, the use of fixed orthodontic appliance was chosen, without including maxillary incisors until there was enough space to position the canines as a result of interproximal stripping in the posterior segment (from mesial surfaces of second premolars to distal surfaces of canines). Treatment planning also included the use of a palatal bar with a Nance holding arch for upper anchorage; extraction of tooth #31 due to gingival recession; and increase in crown height of tooth #41 (0.5 mm distally and mesially).

After the end of conventional treatment, planning included the use of an upper Hawley retainer 24 hours a day during 12 months and during the night for another 12 months. In the mandibular arch, a fixed retainer was bonded on teeth #33 and 43. Special attention should also be given to the process of development of third molars.

## TREATMENT PROGRESS

Treatment began by placing the palatal bar with the Nance holding arch, welded to orthodontic rings attached to teeth #16 and #26, followed by bonding of the fixed appliance (Roth prescription, 0.022 x 0.028-in slot, Straight Wire) in both arches. Mandibular second molars were also included at this stage, differently from maxillary incisors which would be included after canines were properly in place.

The sequence of proximal stripping began with first and second premolars as well as maxillary canines, at high speed, after the interproximal areas of these teeth were separated with the aid of elastic separators. Distalization of first premolars and canines was carried out with elastomeric modules. During the phase of maxillary alignment, there was a need for proximal stripping between teeth #11/21 and 21/22. Alignment and leveling began with NiTi 0.12-in wires up to stainless steel 0.020-in wires. Mandibular archwires were kept by the tie-back method so as to control arch circumference. Maxillary and mandibular rectangular XR archwires were placed before rectangular stainless steel continuous 0.019 x 0.025-in archwires. After crown-root angulation of tooth #41, the patient was referred to have crown height increased with 0.5-mm resin placed on each proximal surface. Treatment finishing included Class II intermaxillary mechanics with elastics and stainless steel 0.018-in continuous archwires for intercuspation.

For retention, a Hawley retainer was used 24 hours a day during 12 months and during the night for another 12 months. In the mandibular arch, a fixed stainless steel 0.028-in retainer was bonded from tooth #33 to #43. As expected, the development and eruption of third molars were monitored.

## RESULTS

**Figure 5.**
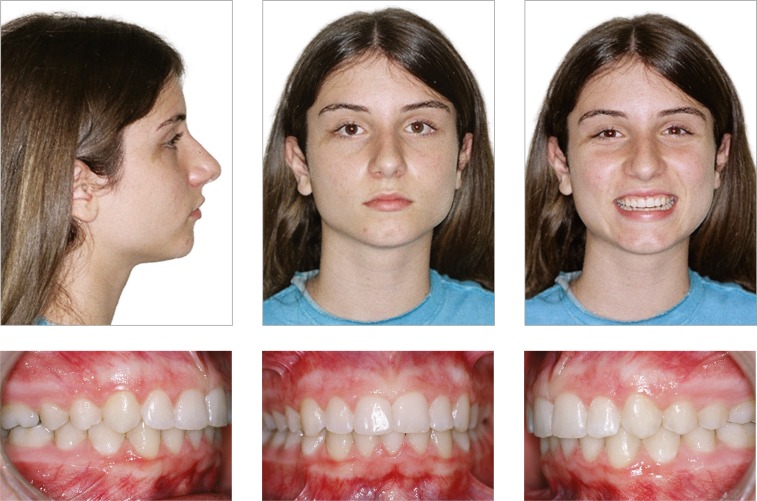
Final facial intraoral photographs.

**Figure 6.**
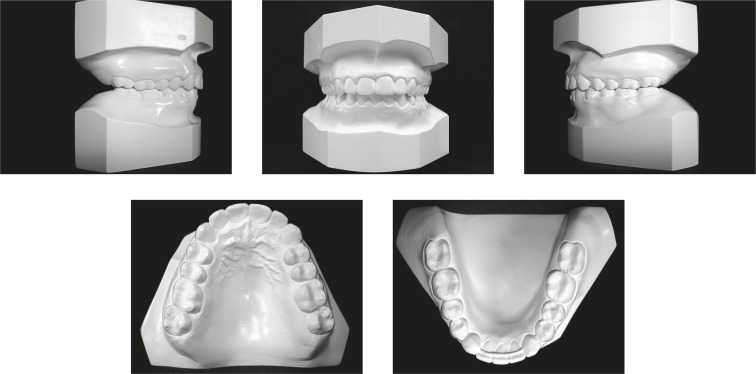
Final casts.

**Figure 7.**
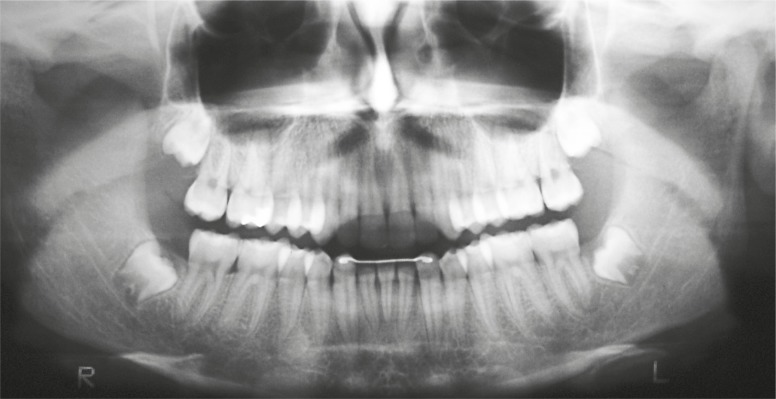
Final panoramic radiograph.

**Figure 8.**
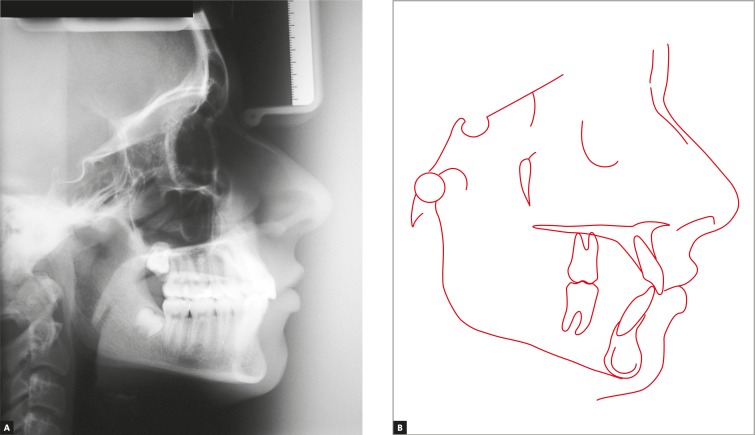
Final lateral cephalogram (A) and cephalometric tracing (B).

**Figure 9.**
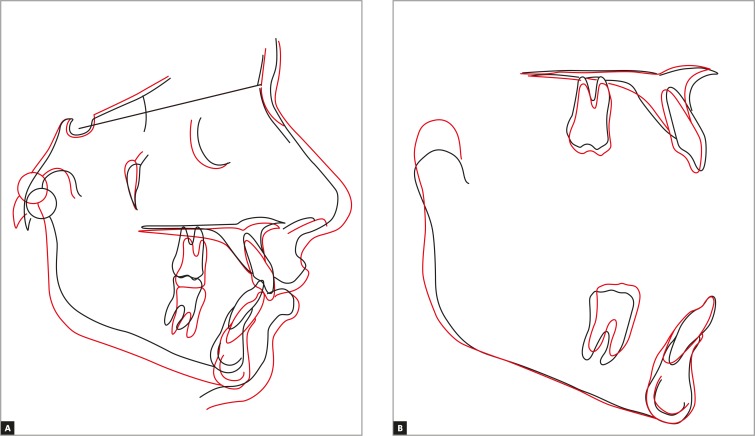
Total (A) and partial (B) cephalometric superimpositions of initial (black) and final (red) cephalometric tracings.

Treatment objectives were reassessed while preparing the final records (Figs 5 to 9) and were found to have been fully achieved. Class I molar relationship remained, while Class I canine relationship and lateral guidance were achieved; maxillary teeth were aligned without significantly changing tipping and protrusion values; mandibular teeth were aligned and leveled; the curve of Spee was corrected; overbite was minimized; and there was an increase in mandibular incisors tipping (3^o^ increase in the 1.NB angle and 6^o^increase in IMPA), but not in protrusion, with reduction of 1 mm in the linear value of 1.NB angle (Fig 9 and Table 1). Additionally, there was a reduction of 0.5 mm in maxillary intercanine width and 1.2 mm in mandibular intercanine width (Table 1).

**Table 1.** Initial (A) and final (B) cephalometric values.

	Measurements		Normal	A	B	Diff. A/B
Skeletal pattern	SNA	(Steiner)	82°	78°	77°	1
	SNB	(Steiner)	80°	73.5°	73.5°	0
	ANB	(Steiner)	2°	4.5°	3.5°	1
	Angle of convexity	(Downs)	0°	8°	5°	3
	Y axis	(Downs)	59°	64°	59°	5
	Facial angle	(Downs)	87°	80.5°	87°	6.5
	SN-GoGn	(Steiner)	32°	35°	33.5°	1.5
	FMA	(Tweed)	25°	28.5°	21°	7.5
Dental pattern	IMPA	(Tweed)	90°	101°	107°	6
	1.NA (degrees)	(Steiner)	22°	22°	23°	1
	1-NA (mm)	(Steiner)	4 mm	5 mm	5 mm	0
	1.NB (degrees)	(Steiner)	25°	30°	33°	3
	1-NB (mm)	(Steiner)	4 mm	7 mm	6 mm	1
	- Interincisal angle	(Downs)	130°	123.5^o^	121^o^	2.5
	1-APo	(Ricketts)	1 mm	3 mm	2 mm	1
Profile	Maxillary lip — S-line	(Steiner)	0 mm	1 mm	-1 mm	2
	Mandibular lip — S-line	(Steiner)	0 mm	3.5 mm	0 mm	3.5

**Table 2.** Measurements of transversal distances of initial (A) and final (B) dental arches.

Measure	A	B	Diff. A/B
Maxillary intercanine width	34.3 mm	33.8 mm	0.5
Mandibular intercanine width	23.4 mm	22.2 mm	1.2

The end esthetic outcomes were highly satisfactory, with the upper midline coinciding with the facial midline and the long axis of tooth #41 (Figs 5 and 6). Passive lip seal and face as well as smile harmony were also achieved. From a dental perspective, the roots of mandibular incisors were parallel with symmetric spaces in between them (Fig 7).

Partial and total cephalometric superimpositions revealed expressive mandibular growth, although reduction in the ANB angle only equaled to 1^o ^(Fig 9 and Table 1).

## FINAL CONSIDERATIONS

Based on the results of this study, it is reasonable to conclude that extracting patient's mandibular incisor contributed favorably to correct mandibular dental crowding which was patient's chief complaint. The minor change caused to mandibular intercanine width can be considered a factor that contributed to increase long-term alignment stability.^1^ Even though there was a potential to increase overjet, this risk was minimized by significant mandibular growth forward (Fig 9). Vertical alveolar growth in the region of mandibular molars favored the correction of both curve of Spee and overbite, without the need for incisors intrusion, which enhanced lower incisors exposure and smile esthetics.^4^ Profile flattening was nonexistent, which could have occurred as a result of premolars extraction.^5^^,^ 6 Mandibular growth reduced facial convexity and favored proper lip seal. 

The clinical case reported herein corroborates the findings in the literature which highlight that extraction of a mandibular incisor might be a good solution to correct mandibular dental crowding,^3^^,^ 7 ^,^ 8 considering the through diagnosis of all issues involved. A set-up might be useful to aid this approach with a view to assessing the implications of extracting a mandibular incisor as well as avoiding an increased overjet.^9^
